# Paracetamol overdose causing acute kidney injury without hepatotoxicity: a case report

**DOI:** 10.1186/s12245-024-00662-w

**Published:** 2024-07-02

**Authors:** Micha Saad, Julien Flament

**Affiliations:** 1 Faculty of Medicine, Université Catholique de Louvain, Avenue Mounier 50, boîte B1.50.04, Brussels, 1200 Belgium; 2 Emergency Department, CHU UCL Namur, 1, rue Dr G. Therasse, Mont-Godinne, Yvoir, 5530 Belgium

**Keywords:** Acute kidney injury, Acute tubular necrosis, Nephrotoxicity, Paracetamol overdose

## Abstract

**Background:**

Paracetamol is a widely used analgesic and antipyretic. Paracetamol-induced hepatotoxicity is well known, but nephrotoxicity without hepatotoxicity is rarely seen.

**Case presentation:**

We present a case of acute kidney injury without hepatotoxicity in paracetamol overdose. A 15-year-old girl was admitted 48 h after she had taken 10 g of paracetamol. She was complaining of abdominal pain and vomiting. Her blood level of creatinine was 1.20 mg/dL on admission, with a peak at 3.67 mg/dL 3 days later. The liver blood tests and blood paracetamol level were negative. She did not receive N-acetyl cysteine and was treated with intravenous fluid (crystalloid). The ultrasonography of the kidneys was normal. Her renal function returned almost to baseline 7 days after admission. It was concluded that the diagnosis was an acute kidney injury caused by acute tubular necrosis due to paracetamol overdose.

**Conclusion:**

This case shows that nephrotoxicity can occur without hepatotoxicity in paracetamol overdose.

## Background

Paracetamol is an analgesic and antipyretic that is in common use worldwide. Paracetamol-related intentional drug overdose among young people has been increasing [1]. Hepatotoxicity is more common than nephrotoxicity. Acute kidney injury (AKI) can occur even in the absence of liver failure, but is a very rare and little-known event [2]. For this reason, we decided to present this case of a 15-year-old girl who presented with acute kidney injury due to paracetamol overdose, without hepatotoxicity.

## Case presentation

A 15-year-old girl with no past medical story was admitted to the emergency department of this hospital because of vomiting.

Two days before this admission she took 10 tablets of paracetamol 1 g (10 g or 175 mg/kg) to kill herself. She told no one that she took this paracetamol. Her neighbor brought her to the emergency department because of 2 episodes of vomiting and epigastric abdominal pain.

On arrival at the emergency department, she told the doctor that she had taken a single ingestion of 10 tablets of paracetamol 1 g 2 days before. Her mother explained that she had found an empty paracetamol 1 g pack in her bedside table, corresponding to 10 tablets. She usually took no medication and did not take other medication like ibuprofen.

On examination, the temporal temperature was 37.3 °C and there were no signs of infection. Vital signs remained stable throughout hospitalization. The weight was 57 kg and the height was 148 cm. The abdomen was painful on epigastric palpation. She had no oliguria or anuria. She had no clinical sign of dehydration.

**Fig. 1:**
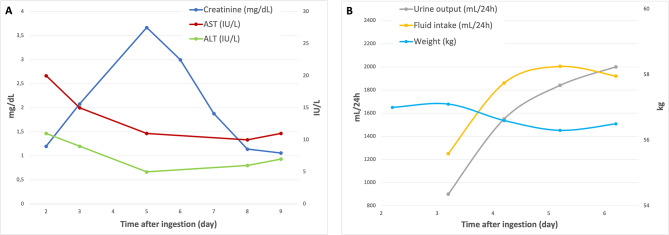
Evolution of blood results, in/out balance and weight. (**A**) The blood creatinine level increased until day 5 after ingestion and returned to almost-normal range on day 9 after ingestion. AST and ALT stayed within normal range for the entire duration of hospitalization. (**B**) Fluid intake represents the sum of oral intake and intravenous crystalloid filling of 1000mL/24 h. The in/out balance was correct and weight remained stable

The blood results are described in Table 1. AST and ALT stayed within normal range for the entire duration of hospitalization (see Fig. 1A.). The blood paracetamol level was < 5.0 mg per liter.

The blood creatinine level was 1.20 mg per deciliter on admission. The peak level of creatinine appeared 5 days after ingestion. The blood creatinine level started to decrease from day 6 after ingestion and had returned to almost-normal range on day 9 after ingestion (see Fig. 1A.). Viral hantavirus serologies were negative. Complements (C3, C4), antinuclear antibody, and anti-neutrophilic cytoplasmic antibodies were negative. Beta-HCG test was negative.

**Table 1:** Blood results during hospitalization

Blood result	Normal values	Day 2 after ingestion (admission)	Day 3	Day 5	Day 6	Day 8	Day 9 (discharge)
Haemoglobin	11.3–15.4 g/dL	13.4	12.4	12.3	13	11.6	13
White cell count x10³	3.90–9.90 /µL	12.82	10.71	7.97	6.03	5.32	5.06
Sodium	137–145 mmol/L	137	141	139	142	143	140
Urea	15–36 mg/dL		33	45	45		
Creatinine	0.52–1.04 mg/dL	1.20	2.08	3.67	3	1.14	1.06
Total bilirubin	0.00-1.30 mg/dL	0.51	0.4				
Aspartate transaminase	5–40 IU/L	20	15	11		10	11
Alanine transaminase	5–40 IU/L	11	9	5		6	7
C-reactive protein	< 5 mg/L	21.54	27.95	41	33.86	13.18	8.18
Paracetamolemia	< 5 mg/L	< 5					

The spot urine sample results are described in Table 2. Based on these urine analyses, the fractional excretion of sodium was 0.3% on admission and 1.2% on day 5. The fractional excretion of urea was 35.8%. The 24-hour urine sample results are described in Table 3.

**Table 2:** Spot urine sample results

Spot urine sample	Day 3	Day 8
pH	5	
Culture	Negative	
Erythrocytes (< 13/µL)	14	
Leukocytes (< 13/µL)	17	
Granular casts	+	
Protein (0–0.100 g/L)	1.670	
Protein: creatinine ratio (< 20)	0.97	
Microalbumin (< 17 mg/L)	234.94	
Beta-2 microglobulin (0.0-0.15 mg/mL)	0.33	
Osmolality (mOsmol/kg)	311	
Sodium (mmol/L)	25	87
Urea (mg/dL)	379	721
Creatinine (mg/dL)	66.78	57

**Table 3:** 24-hour urine sample results

24-hour urine sample	Day 5 (after ingestion)
Sodium (40–220 mmol/24 h)	17
Urea (26–43 g/24 h)	3
Creatinine (0.6–1.6 g/24 h)	0.6
Creatinine clearance (mL/min)	12
Protein (< 0.15 g/24 h)	1.18
Oxalate (< 50 mg/g creatinine)	16.70

An ultrasound study of the urinary tract revealed no abnormality and no obstacle in the urinary tract.

She was given intravenous crystalloid fluid therapy with a flow rate of 1000mL/24 h for 4 days (See Fig. 1B.). No N-acetyl cysteine (NAC) was administered. Psychological therapy was initiated during hospitalization and continued outside the hospital. After 7 days of hospitalization the patient was discharged and recovered well.

## Discussion

In paracetamol overdose, nephrotoxicity is less common than hepatotoxicity. Acute kidney injury can occur even in the absence of hepatotoxicity [3]. The incidence of isolated acute kidney injury (AKI) is unclear. It is very rare, ranging from 0.4 to 1.25% [2]. Many studies have demonstrated that large doses of paracetamol can cause kidney injury in rodent models [4].

Paracetamol is metabolized approximately 63% via glucuronide conjugation, 34% by sulfate conjugation, and less than 5% is oxidized by the cytochrome P-450 enzyme system to form N-acetyl-benzoquinone imine (NAPQI), which is a reactive intermediate. These phases occur in the liver and metabolites are excreted in urine. When a therapeutic dose is ingested, NAPQI is then reduced by glutathione and also excreted in urine. In paracetamol overdose, NAPQI accumulates to a level that exceeds the capacity of glutathione to neutralize it [5, 6]. The mechanism of liver failure due to paracetamol overdose is well known. NAPQI can initiate free radicals or bind covalently to macromolecules, leading to cell damage with consequences ranging from mild disturbance of liver enzymes to fulminant hepatic failure [3].

In contrast to the hepatotoxicity, the mechanism of the nephrotoxicity remains unclear. In nonhuman animals, paracetamol overdose causes marked glutathione depletion in the liver, but not in the kidney [7]. Other animal studies indicate that paracetamol oxidation by the cytochrome P-450 system may result in tubule damage, which can be potentiated by glutathione depletion in the kidney [8]. The mechanism underlying paracetamol’s nephrotoxicity may be the induction of apoptosis through caspase activation [7, 9]. Paracetamol induces apoptosis in cultured murine tubular epithelial cells, probably by increasing endoplasmic reticulum stress [9].

NAC is a glutathione precursor that helps to restore glutathione and neutralizes reactive intermediates. Although the role of NAC has been demonstrated in the prevention of severe hepatotoxicity, there is no benefit to administered NAC in preventing nephrotoxicity [6]. This is another argument supporting the hypothesis that the mechanisms of nephrotoxicity and hepatotoxicity could be different.

Our patient did not receive NAC therapy on admission because the liver blood tests and blood paracetamol were negative.

A renal biopsy is rarely performed, but the histology of biopsied cases reflects an acute tubular necrosis (ATN) pattern in a study conducted on murine [9]. In the case of ATN, the urinalysis shows granular casts with variable hematuria or pyuria. The urine sodium tends to be > 40 to 50 mmol/L due to tubular injury [10]. The urine osmolality is similar to that of plasma [5, 6]. Increased urinary beta-2 microglobulin and blood gamma-glutamyl transferase could be markers of early paracetamol nephrotoxicity [7]. In this case, we did not perform a biopsy. The kidney function improved on its own, so biopsy would not have changed the treatment or prognosis, and it is an invasive technique. The urine sodium was 25 mmol/L on admission but rose to 87 mmol/L on day 8 and there was an increase in urinary beta-2 microglobulin. The blood osmolality was missing.

AKI with paracetamol overdose is more common in adolescents and young adults, but the reason is unclear [6]. Isolated AKI has been observed to occur with an average age of 18.8 years [2]. An explanation could be that endoplasmic reticulum stress decreases with age. Thus, the stress response is stronger in younger people than in older people. If this trend also exists in the kidney, the response could be stronger, leading to more severe apoptosis and more nephrotoxicity in younger people [11].

Paracetamol intake has a higher risk of toxicity in patients who consume alcohol, in case of starvation or fasting, and in persons who take medications that induce the P-450 microsomal oxidase enzyme [5, 6]. Isolated AKI, in contrast to combined hepatotoxicity and nephrotoxicity, appears to occur when a lower dose of paracetamol is ingested [2]. Our patient was young, took a small dose of paracetamol (10 g), and had none of these risk factors.

In most cases, AKI appears between 2 and 5 days post ingestion, with peak levels of creatinine at an average of 7 days post ingestion. Baseline renal function tends to return within 1 month [6]. A significant rise in serum creatinine concentrations might not be detectable until more than 2 days after ingestion, nephrotoxicity can therefore easily be missed if the patient returns home within 48 h [7]. In this case, AKI appeared 2 days post ingestion, with peak levels of creatinine 5 days post ingestion. The creatinine started to decrease at day 6 post ingestion and recovery with fluid challenge. Our patient’s renal function decreased, then returned to baseline 9 days post ingestion. Thus, if the patient had presented immediately to the emergency department after her intentional drug overdose, the AKI might not have been diagnosed.

In this case, acute tubular injury due to paracetamol seems to be the likely cause of AKI. There was no evidence of dehydration in our patient. Kidney ultrasound excluded a post-renal cause of AKI. Viral serological and autoimmune blood tests were negative.

## Conclusion

In summary, paracetamol nephrotoxicity without hepatotoxicity is not uncommon and patients sould be monitored for over 48 to 72 h. Although the role of NAC is well known in hepatotoxicity, its role has not been demonstrated in preventing nephrotoxicity.

We should keep in mind that when patients present immediately after paracetamol overdose, acute kidney injury may be delayed and without accompanying hepatotoxicity if a small dose was taken, especially in young people.

## Data Availability

Please contact author for data requests.

## Abbreviations

AKI: Acute kidney injury
AST: aspartate aminotransferase
ALT: alanine aminotransferase
NAC: N-acetyl cysteine
NAPQI: N-acetyl-benzoquinone imine
ATN: acute tubular necrosis
